# Supplementary figures and images for: The potential virulence of Listeria monocytogenes strains isolated from fresh produce processing facilities as determined by an invertebrate Galleria mellonella model
Source: PLoS One. 2024 Dec 12;19(12):e0311839. doi: 10.1371/journal.pone.0311839 (PMC11637379; doi:10.1371/journal.pone.0311839)

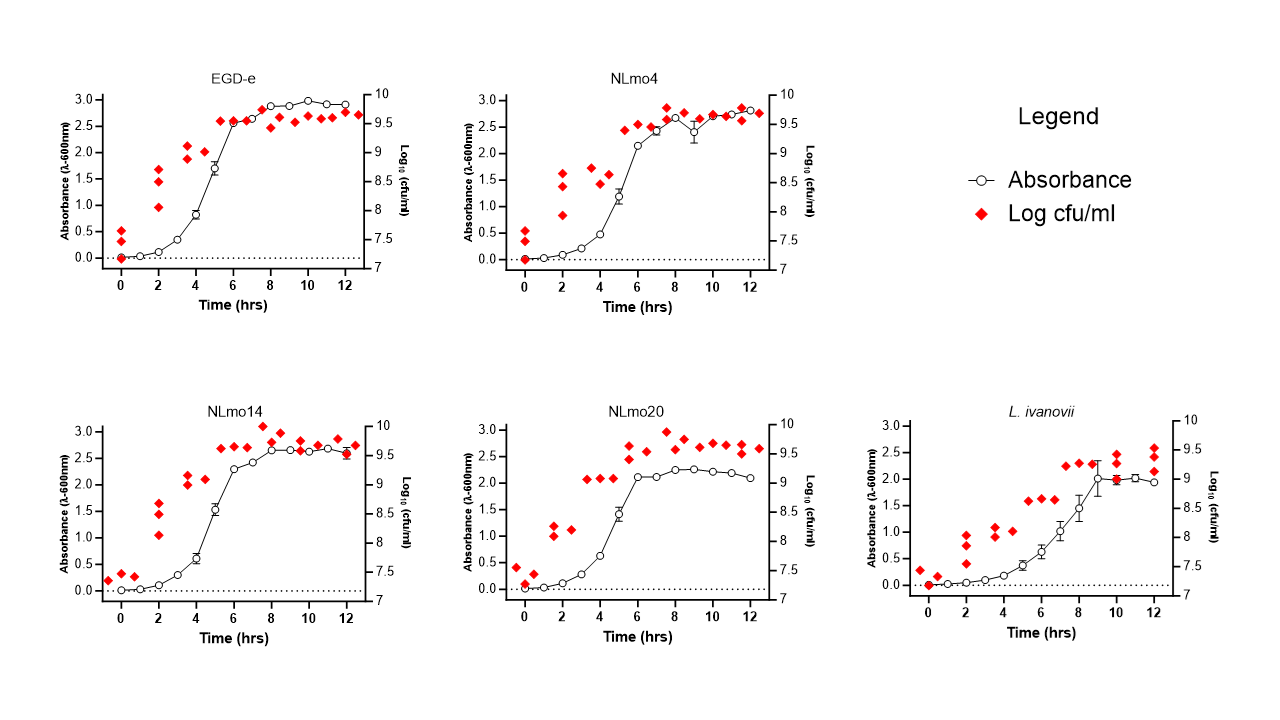

Supplement: S1 Fig — BHI broth was seeded with bacterial cultures in stationary phase to a starting absorbance (OD60nm) of 0.01 and incubated at 37°C (200 rpm, aerobic conditions). Absorbance was measured hourly and CFU ml-1 at bi-hourly intervals. Results represent individual replicates (CFU ml-1) and mean ± SEM values of three independent determinations. (TIF) [file pone.0311839.s001.tif]
