# Supplementary material for: The potential virulence of Listeria monocytogenes strains isolated from fresh produce processing facilities as determined by an invertebrate Galleria mellonella model
Source: PLoS One. 2024 Dec 12;19(12):e0311839. doi: 10.1371/journal.pone.0311839 (PMC11637379; doi:10.1371/journal.pone.0311839)
Supplement: Attachment — Submitted filename: Response to reviewers.pdf [file pone.0311839.s002.pdf]

Edinburgh Napier
UNIVERSITY

School of Applied Sciences
Room 2.B.45, Sighthill Campus
Edinburgh Napier University
EDINBURGH, EH11 4BN
n.wheelhouse@napier.ac.uk

11th Sept 2024

The Editor, PLOS ONE

Dear Sir/Madam,

We are grateful for the reviewers and editorial offices' prompt and meticulous reviews of our manuscript. We enclose the modified manuscript and we hope we have satisfactorily made the necessary amendments for publication.

Editorial requirements

1) We have made changes to the fonts and layout including header fonts to meet PLOS ONE's style
2) We have amended the acknowledgements section and removed the funding information.
   Please amend the Funding statement 'The project was funded through a Edinburgh Napier University Research Excellence Grant'
3) We have removed reference to 'data not shown'
4) We have added the 'Supporting Information' caption to the end of the manuscript

Reviewer #1: This study aimed to evaluate the virulence of several strains utilizing the Galleria mellonella (G. mellonella) invertebrate infection model, which underscores the importance of delving deeper into the research on Listeria monocytogenes.

(1) When Listeria monocytogenes first appears in the abstract and main text, it should be written in full. Subsequent mentions can utilize the abbreviated genus name.

Response: We thank the reviewer and apologise for the oversight. The appropriate changes have been made throughout the document

(2) Regarding the choice of serotype EGD-e, it would be beneficial to clarify the rationale behind this selection. Current research frequently references molecular typing such as ST8, ST87, and ST9, which are known pathogenic types. While the abstract mentions "little is known about the potential virulence of Listeria isolates from

the fresh produce environment" and the introduction states "to determine the virulence potential of isolates of the bacterium in order to evaluate the human health risks they pose," these aspects are not extensively discussed in the introduction, especially in relation to molecular typing. Incorporating a discussion on why a serotype-based approach was not taken, given its close association with molecular typing, would strengthen the methodology section.

Response: We thank the reviewer for their comments. In terms of approach we have stated in the introduction that we were investigating the virulence potential of a number of isolates from the food industry which had been previously characterised by molecular typing and WGS (Smith et al, 2019). The study was not to compare methods of typing and in the context of the paper we are not sure that a discussion on a serotype approach would be appropriate. In terms of EGD-e it was used as it is a well characterised and extensively used strain, we have added this clarification (Line 82) citing the relevant reference Becavin et al, 2014.

(3) More detailed information and analysis regarding the HISS index should be included. The basis for its selection and how it contributes to the overall assessment of virulence need to be elaborated upon.

Response: We have extended the section and added addition appropriate text to highlight the relevance of the HISS (Line 124-132)

(4) Justification for the inclusion of Listeria ivanovii and further analysis specific to this species should be provided. Explaining its relevance to the study and how it informs our understanding of virulence mechanisms would enrich the discussion.

Response: L. invanovii is a related organism that demonstrates significantly lower levels of pathogenicity. We have included a sentence in the methodology (Line 84-87) to clarify this point

(5) The discussion should address how existing research has facilitated the development of Dose-Response models and risk assessment studies. Highlighting specific advancements or gaps that this study aims to fill would demonstrate the novelty and significance of your work. Furthermore, discussing the potential implications of your findings for public health policy and risk management strategies would enhance the impact of your research.

Response: We thank the review for their comments. We have incorporated additional text in the discussion to highlight the relevance of this work to risk management (Lines 482-487)

Reviewer #2: This paper was based on rigorous academic standards. The introduction provided the necessary background information.

The research methodology for the study is appropriate. However, statistical analyses in material and method section were absent. It should be

Response. We thank the reviewer for their comment and have added the appropriate section (Lines 168-174)

Thank you for your time and consideration.
Yours sincerely,

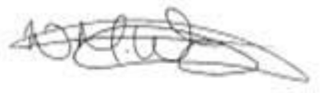

Dr Nick Wheelhouse
Professor in Microbiology
Edinburgh Napier University
